# Listening to women’s voices: the experience of giving birth with paramedic care in Queensland, Australia

**DOI:** 10.1186/s12884-019-2613-z

**Published:** 2019-12-20

**Authors:** Belinda Flanagan, Bill Lord, Rachel Reed, Gail Crimmins

**Affiliations:** University of the Sunshine Coast, ML 40, Locked Bag 4, Maroochydore DC, QLD 4558 Australia

**Keywords:** Birth before arrival, Unplanned, Narrative inquiry, women’s perspective

## Abstract

**Background:**

Unplanned out-of-hospital birth is generally assumed to occur for women who are multiparous, have a history of a short pushing phase of labour or are experiencing a precipitate birth. However, there is little research that examines the woman’s perspective regarding factors that influenced their decision on when to access care. This research aimed to explore women’s experience of unplanned out-of-hospital birth in paramedic care. Due to the size of the data in the larger study of ‘Women’s experience of unplanned out-of-hospital birth in paramedic care’ [1], this paper will deal directly with the women’s narrative concerning her decision to access care and how previous birth experience and interactions with other healthcare professionals influenced her experience.

**Method:**

Narrative inquiry, underpinned from a feminist perspective, was used to guide the research. Twenty-two women who had experienced an unplanned out-of-hospital birth within the last 5 years in Queensland, Australia engaged in this research.

**Results:**

The decision of a woman in labour to attend hospital to birth her baby is influenced by information received from healthcare providers, fear of unnecessary medical intervention in birth, and previous birth experience. All themes and subthemes that emerged in the women’s narratives relate to the notion of birth knowledge. These specifically include perceptions of what constitutes authoritative knowledge, who possesses the authoritative knowledge on which actions are based, and when and how women use their own embodied knowledge to assess the validity of healthcare workers’ advice and the necessity for clinical intervention.

**Conclusions:**

The women interviewed communicated a tension between women’s knowledge, beliefs and experience of the birth process, and the professional models of care traditionally associated with the hospital environment. It is essential that information provided to women antenatally is comprehensive and comprehensible. The decisions women make concerning their birth plan represent the women’s expectations for their birth and this should be used as a means to openly communicate issues that may impact the birth experience.

## Background

An unplanned out-of-hospital birth or what is commonly referred to as a ‘Birth Before Arrival’ or ‘BBA’, is a common term referring to a planned hospital birth of a baby ≥20 weeks gestation and > 400 g, that occurs either at home or en-route to a hospital or birthing centre facility [2]. In many cases an ambulance is called to assist with the birth. However, in some situations the woman may be on her own, at home or in a vehicle, with the potential of being a significant distance from medical assistance.

In Australia, most births occur in a hospital (97%) and only a small portion are planned to occur in a birthing centre (1.8%) or home (0.3%), yet most recent statistics report that in 2016, 1237 births occurred prior to arrival at hospital; these account for 0.4% of all Australian births [3]. The greater majority of unplanned out-of-hospital births occur in multiparous women or women experiencing a precipitate birth, however, factors such as geographical distance from a maternity unit and low socio economic status, have also been identified [4–6]. An unplanned out-of-hospital birth can present with significant physical and psychological risks [5, 7] and the ability of healthcare providers to identify women at risk is problematic [8, 9].

Whilst some claim that the responsibility for an unplanned out-of-hospital birth occurring lies with the women [9–11], there are wider social and organisational issues that impact childbirth planning. For instance, findings from the Australian Maternity Services Review reveal that many consumers are concerned that organisational issues influence the quality of maternity care provided to Australian women. More specifically, women identified a limited availability of models of care consistent with their expectations; negative birth experiences and the impact this had on themselves, their babies and their families; difficulties in sourcing information and making informed choices on maternity care; perceptions of risk; and, for many, their desire that pregnancy and birth be seen as a natural process [12].

There is a paucity of literature that explores women’s perspective concerning their decision to access care when in labour and their experience of unplanned out-of-hospital birth. Existing literature reports the reasons for births occurring in unplanned and unexpected ways [9] and also debates the increased maternal and neonatal morbidity and mortality rates [5, 7, 13, 14]. Others, who specifically explore the women’s experience of birth look mainly at the psychological impact of the event on the mother [15]. These also are not from an Australian perspective and occur in countries with a different system of maternity care. Yet, there are many social and organisational aspects to birthing in Australian maternity systems that may impact a labouring woman’s decisions to access maternity care. To more comprehensively understand the phenomenon of unplanned out-of-hospital birth it is important to explore the women’s perspective. The aim of this study is to explore the women’s experience of unplanned out-of-hospital birth in Queensland, Australia. This study is part of a larger study that explores the experience of unplanned out-of-hospital birth in paramedic care [1], therefore each woman birthed with paramedic attendance.

## Methods

This study used semi-structured interviews, conducted and analysed using a narrative inquiry framework from a feminist perspective, to explore women’s experience of unplanned out-of-hospital birth in paramedic care.

### Learning through birth stories

Narratives of birth stories can provide rich descriptive data and allow researchers the opportunity to present the experience holistically [16, 17]. In the context of our research, birth stories were a reliable and credible form of data. The told story presented a faithful description of how the women recalled their birth experience emotionally and cognitively. To ensure validity, the woman’s own words were crucial in interpreting the study findings. Placing the woman at the centre of understanding and knowledge development privileges a feminist approach and allows women’s narratives to contribute to a midwifery body of knowledge.

Narrative inquiry is aligned with feminist research because the woman and her interpretation of the experience is fundamental to her life story. In this research, it was important to undertake a critical perspective by examining the woman’s interactions and actions. Feminist criticality in this context includes an evaluation of the verbal and non-verbal communication and practice, as well as routines and habits that are embedded with psychosocial structures of power and authority [18].

### The recruitment process

The sampling of recruited participants was purposeful and allowed for only those that have experienced unplanned out-of-hospital birth in paramedic care to be recruited. To ensure participants had experiences that were relevant to the research aims specific inclusion criteria were identified. Participants were required to be:

- English speaking;
- over the age of 18 years at the time of the interview;
- birthed in the state of Queensland, Australia within the previous 5 years; and had an unplanned out-of-hospital birth in paramedic care.

Recruitment and data collection occurred in 2017 and was achieved by advertising for participants utilising social media, on-line and print newspapers. According to Ireland & van Teijlingen [19], purposive sampling involves continued recruitment and interviewing until no further themes arise. This requires continuous analysis to recognise emerging themes and identify when saturation is reached. Recruitment was discontinued at 22 interviews as no further themes were identified.

### Data collection

The narrative interview was conducted in a one-to-one environment by the principle researcher who has a clinical background of midwife and paramedic. Each interview was 45 min to 1 h in duration. All women participating in this study gave a verbal account of their birth experience. The narrative interviewing process began with a single framing question: “*Can you tell me about your experience of childbirth*?” The women were thus invited to describe the experience as deeply as possible using narrative to express their feelings and thoughts. This depth is necessary to understand the experience; memories conveyed as stories retain the contextualised complexity of the situation. It was occasionally necessary to ask more pointed questions to allow for the clarification of specific aspects of the narrative; for example, “*How did your experience compare with your expectations of birth?”* Or *“Looking back would you say that the experience has changed the way you see yourself and your views on birth?”*. Personal stories were collected with holistic perspectives, not exclusive stories of the birth event.

Maintaining rigour within the narrative inquiry process was important to both validate the credibility of the findings and to create a research trail so that the research stages followed were easily understood. To ensure the quality of this research a ‘members check’ or ‘members validation’ was used whereby participants were invited to offer further comment, expand on, or clarify issues that were considered important to them in the week following the initial interview and prior to transcription. Further comments and clarifications from participants were incorporated into interview transcripts for analysis. To achieve confirmability or auditability, which refers to the efficacy of the information reported, field notes, transcripts, audio recordings, email correspondence, data reconstructions and data synthesis were included in an audit trail.

### Analysis

Thematic analysis was undertaken using the six phases of qualitative analysis recommended by Braun and Clarke [20]. Phase 1 - Familiarising yourself with the data - This step requires the researcher to be fully immersed and actively engaged in the data. Phase 2 - Generating initial codes - identify preliminary codes. Phase 3 - Searching for themes - interpretive analysis of the collated codes. Phase 4 - Reviewing themes - A deeper review of identified themes. Phase 5 - Defining and naming themes - This step involves refining and defining the themes and potential subthemes within the data. Phase 6 - Producing the report - transform the analysis into a report using extract quotes that relate to the themes.

NVivo software [21] was used to organise and analyse the data. However, the feeling and emotional depth of the narrative telling couldn’t be identified by NVivo. Listening to the audio and reading the transcribed accounts provided a sense of closeness to the information conveyed in the interview. This allowed for displays of humour, disappointment, sadness and irony which were lost using computer assisted analysis. Due to the limitations of NVivo and to further immerse into the narrative, more practical forms of analysis were used. Listening to each audio recording and formulated a short paragraph to describe the main narrative, punctuating the main narrative with seemingly significant quotes and noting any strong feelings that were expressed by the participant. This allowed for circumstances outside the original themes to be acknowledged and valued. The result of the data analysis was an explanation of the event that was retrospective.

### Overall ethical considerations

Ethics approval was provided by the University of the Sunshine Coast Human Research Ethics Committee (HREC: S/15/825). Participants were provided with a Research Project Information Sheet which described the purpose and process of the intended research project. They were also assured that withdrawal from the research project could be initiated at any time and follow-up with a perinatal psychologist was available upon request. Participants were informed that their interview would remain confidential and de-identified at the time of transcription.

## Results

### The participants

Participants’ age ranged from 20 to 42 years at the time of the interview. At the time of the unplanned out-of-hospital birth participants were between the ages of 16–39 years; four mothers were primipara and 18 were multigravida. Geographically, participants were from various locations (city, inner and outer regional) in the state of Queensland and were originally booked for a hospital birth in a public or private hospital or a birth centre. All mothers had received the minimum Queensland standard of recommended antenatal care [22].

### Birth knowledge

**Fig. 1:**
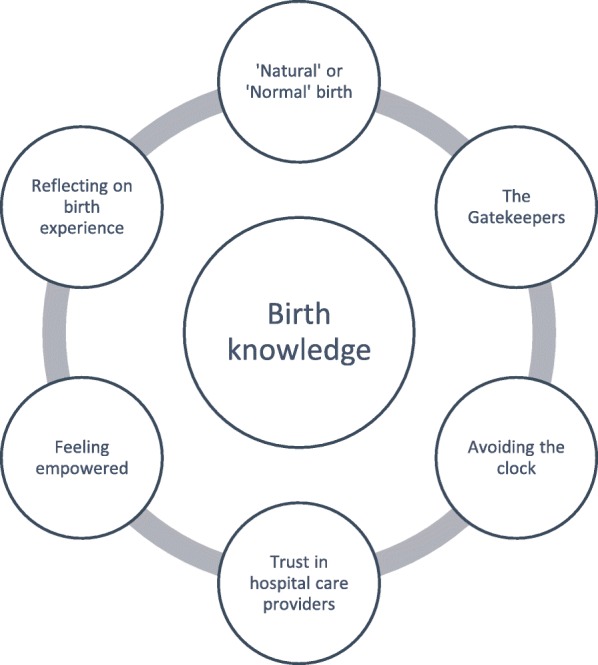
Themes and subthemes

The overarching theme that emerged from within the women’s narratives was ‘Birth Knowledge’ and from this several sub-themes were identified (Fig. 1).

Women referred to two types of birth knowledge: Authoritative Knowledge – the knowledge they perceived as important, relevant, and consequential for decision-making [23]; and Embodied Knowledge - information our bodies know and use without conscious thought, otherwise known as Instinctive Knowledge. Women’s narratives revealed the role birth knowledge played in the woman’s ability to exert agency during the unplanned out-of-hospital birth experience and the ways in which healthcare professionals contributed to authoritative knowledge.

### The quest for ‘natural’ or ‘normal’ birth

Many women in this study, both multipara and primipara, identified a need for what they described as a ‘natural’ and ‘normal’ childbirth with varying degrees of medical intervention. In their pursuit for a ‘natural’ childbirth, women described being ready to inform the healthcare professional of their preference by writing specific birth plans or entering the institution with a previously established set of rules that would allow for what they perceived to be the requirements for a natural birth.

*Participant 8 - I didn’t want any vaginal examinations because I didn’t want the hospital knowing I was ten centimetres and then giving me one hour to push... I was going to refuse all vaginal examinations. No pain relief, no drugs at all … I wanted delayed cord cutting and I wanted immediate skin-to-skin contact and breastfeeding after the birth. I did get all that just with paramedics, no hospital.*

Women also discussed their previous birth experience and interactions with healthcare professionals, how they felt disadvantaged by a medicalised model of childbirth, and experienced pressure to negotiate against certain medical interventions:

*Participant 5 - I declined a number of tests … throughout my pregnancy … After that first birth experience I become a bit anti-interventions and monitoring … I became a little bit more vocal … I was going to be a lot more insistent that things be done the way I wanted.*

Even though women had conceptualised a personal set of rules that would provide for what they considered to be a natural birth, some indicated that they feared they would be coerced or manipulated to conform to institutional policy that was in contradiction to their plan. The solution for some was hospital avoidance; they decided that it was in their best interest to stay at home for as long as possible to protect themselves against medical interference.

*Participant 8 - I guess that my strategy with the second birth was to avoid going to hospital for as long as I could... to sort of wait until I was in transition so I could spend as little amount of time in the hospital in labour as possible so there’d be less chance of an intervention.*

All multipara women indicated that there were many obstacles to them giving birth naturally in their preferred location, medical intervention being the most dominant. To protect themselves from medical intervention some women declined or avoided hospital until labour was established. Many women in this study acknowledged that they would experience barriers to them having a natural birth in a hospital.

*Participant 18 - I’m not actually listening to what you’re [doctors and midwives] saying, I’m not taking on board what you’re saying because it doesn’t have to be like that. They were like, just get your drugs straightaway because that’s what’s become the normal.*

### The gatekeepers

Women discussed authoritative knowledge in relation to the triage process of admitting to hospital women in labour, which in Australia usually encompasses three stages: (i) women are encouraged to contact a birthing facility via telephone to allow a midwife to determine if active labour has commenced; (ii) the woman may be invited to present to the birthing facility to be physically assessed, without guarantee of being ‘allowed’ to stay; and (iii) if the midwife determines the woman is in active labour, as defined by Queensland Health policy [24], the woman will be admitted. The term ‘gatekeepers’ is used by the authors to describe midwives’ and doctors’ authority or ‘power’ in determining labour and ‘allowing’ admission to the birthing facility.

Primipara participants communicated their difficulty in identifying with the hospital’s definition of active labour. Some were confused about when labour commenced as they tried to interpret what they were feeling and how it fit into the definitions of Braxton Hicks, early labour or active labour.

*Participant 7 - I did get pretty decent Braxton Hicks from about sixteen weeks … that actually ended up being quite painful and breathtaking … When my labour actually started, I couldn’t really tell the difference … I’d been I guess, in hindsight, what’s called early labour most of the day.*

Both multipara and primipara women identified a concern about attending hospital at the ‘right time’ to avoid being sent home. This concern stemmed from messages they had received from midwives during antenatal care.

*Participant 7 - The reason this happened, giving birth at home, was because I felt that it was drummed into us that much in the antenatal classes that it won’t happen quickly and that you’ll be in labour for hours and hours. They told you … the average labour … and that you would be in each phase for hours with it being your first and … don’t go to hospital too early because you’ll get turned around and sent home.*

Being assessed as ‘not in labour’ and sent home caused the women to feel unsupported and anxious about ‘getting it wrong’ if seeking care too early.

*Participant 12 - We’re told … don’t come in until you’re getting the decent contractions because you don’t want to be sent home having done nothing.*

Participants also described feeling vulnerable and unsupported when appealing to midwives to allow them to stay once they had presented in early labour.

*Participant 18 - She said to me you’re not in labour … you’re not in full blown labour, this is just Braxton Hicks contractions … As she was saying this to me, I was just lying there bawling my eyes out because … no one was listening to me.*

The participants expressed that their perception of active labour was not validated by hospital staff when they presented to hospital or were triaged over the phone and felt inferior and undermined. These women’s stories offered a counter narrative to the medical ‘authoritative’ dominant discourse. As a counterpoint, some women felt they possessed an authoritative embodied knowledge which, because it appeared misaligned with that of the medical authority, caused them to delay presenting to hospital.

*Participant 18 - How can you judge [labour progress] … because everyone’s different, everybody’s different? Does that make sense? That it’s just like everyone’s a cookie cutter and one thing works for everyone. It doesn’t work for everyone.*

### Avoiding the clock

Women described how they were aware of hospital policy concerning time schedules for the ‘normal’ progression through labour and birth, and the possibility of medical intervention to speed up labour if they did not meet such expectations. They also communicated that they were well informed of interventions such as being put ‘on the clock’ when admitted to hospital because of education they had received from midwives, other women, or their own previous birth experience. The concept of women being pressured to accept early intervention to progress labour and putting themselves at risk was a prominent message the participants had received.

*Participant 12 - My midwife and I discussed about staying at home as long as I could so that when I came to the hospital … I was well and truly in labour. I know we tell women that if they come in earlier the more likely they are to have the interventions. I know for myself it was important for me to stay home as long as I could.*

Although it was communicated that some midwives tried to protect the mother from ‘the clock’ and hospital policy, some women felt a need to protect themselves by staying at home as a way of avoiding being coerced into interventions.

*Participant 6 - I wanted to stay at home as long as I could … that way it was going to prevent intervention... They [midwives] said, “If you go to hospital and then you’re not progressing they’re probably going to start pushing for you to have intervention.” And I didn’t want that, so I was quite happy to stay at home … It’s your own birth but you don’t get much of a say because the hospital has its guidelines that it follows... that’s why we were trying to stay at home and not go to the hospital too early …*

Most of the participants felt they would be deprived of birth choice and agency when they entered the hospital system. The women suggested that their ‘voice’ and autonomy would be taken away from them and medical intervention would be more likely to occur.

*Participant 8 - It’s funny, isn’t it? That you have to lie about things or that you have to be incredibly assertive to get the kind of birth that you want, and you shouldn’t be under that sort of pressure in that sort of setting.*

### Trust in hospital care providers

Based on previous birth experiences, some women expressed a lack of trust in the care provided by midwives and doctors. Participants described being treated poorly and feeling unsupported. They suggested that midwives have lost their way and the concept of ‘being with woman’ during the childbirth was no longer important to them.

*Participant 8 - They (midwives) don’t have a lot of understanding of a natural process of a birth. They are only going by what they know and what they’ve been taught, and I think they’ve been taught the wrong way … there are so many women that are being traumatised and going through everything in such a clinical, uncomfortable, horrible way and thinking that’s the only way.*

Some women communicated negative feelings about midwifery care, either in relation to previous births or during admissions to hospital prior to their unplanned out-of-hospital birth. Specifically, some participants commented that they felt judged by midwives or, based on previous birth experience, did not trust midwives to advocate for them.

*Participant 22 - I don’t think there’s enough midwives practising that … give women other options. I know there’s some great ones but my experience in the hospital was not positive. The midwives were not helpful, it was pretty bad.*

Although some participants expressed these feelings of protecting themselves from intervention, policy and the midwives themselves, two women communicated a strong connection with their midwife.

*Participant 11 - I feel like midwives’ rock because they’re like your second brain … they hold all your content … I feel like [the midwife is] … my brain outside of my body … that’s telling me the things that I wanted to happen because I’m not in the front mind frame to be able to make those decisions for myself.*

However, positive anecdotes about midwifery and medical care did not emerge as common themes in this research. Women who commented on previous birth experience predominantly reported negative interactions with hospital staff and a lack of support in fulfilling their desired and clearly expressed birth plan.

### Ways of knowing

The experience of ‘knowing’ for a pregnant woman, has both social and cultural associations, and includes cognitive, intuitive and embodied knowledge [25]. Some women in this study trusted their embodied or instinctive understanding more than the scientifically grounded knowledge received from healthcare professionals:

*Participant 21 - I think partly because of medical intervention there’s a lot of issues too and that women don’t trust in their bodies, or maybe aren’t given the opportunity to trust their bodies.*

Thus, the women in this study valued multiple ways of knowing, including intuitive or embodied knowledge. Many women discussed non-rational ways of knowing as important to them during the birth process.

*Participant 12 - It just reiterated the fact that women’s bodies are amazing, and childbirth is normal and we were actually made to do that … your body’s actually really intuitive as to what’s happening … just go with it.*

In addition, some women claimed that their intuition was a legitimate and authoritative form of knowledge. As labour progressed and the pain increased many women reported that they became less panicked, more aware of what was occurring and were increasingly reliant on their birthing instincts. They described transitioning from a rational mind set to a more instinctive one***.***

The participants were confident in their body’s ability to birth without assistance, and this confidence concerning childbirth positively affected how well they coped physically and how they felt about the experience emotionally.

*Participant 2 - I have quite a fearless attitude towards birth as well. So, I’ve never had any fear around having a baby...I would have been fine to birth the baby myself in the home or on the side of the road if that had happened … I had the confidence that my body could do it and that everything was going to be okay.*

Women’s narratives did not often express any significant concerns that something might ‘go wrong’ without medical intervention. This is counter to the findings of Erlandsson and colleagues [15] in a similar study in Sweden. In this study the women instead held an intuitive understanding that if they trusted themselves and their bodies, they would be safe, and their child would be born without complication. This view opposed the care providers’ authoritative knowledge that indicated a lack of trust in women’s bodies and determined that ‘safety’ could only be created from intervention.

### Feeling empowered

All the women in this study experienced self-confidence and trust in what their bodies could achieve during pregnancy and birth. They described feelings of exhilaration and power and an ability to take charge of the birthing situation, regardless of its unplanned and unexpected nature. The result was a sense of mastery, satisfaction, and the discovery of inner strength.

*Participant 12 - I felt pretty pumped afterwards. As soon as like he was born, and we were going out to the ambulance I said to my husband let’s do that again...I felt really pumped … Proud of the job I’d done... I come from a line of women needing assistance with having babies, Caesars and things like that. The fact that I haven’t had to have a Caesar and that it just felt good. It felt really quite amazing... I felt that bit more empowered by it, the fact that it didn’t go to plan, but it was pretty awesome still.*

Some women felt so empowered that the experience changed their plans for future births.

*Participant 8 - In the end, it was quite lovely, and it was lovely that my son was there and it was lovely it was at home. I mean, overall it was such a positive experience and especially compared with the first birth that if I have a third child …*. *We’ve already discussed it and we will definitely have a home birth.*

### Reflecting on previous birth experience

Some women drew comparisons between their unplanned out of hospital birth with previous birth experience. Some spoke of traumatic previous birth experiences that they describe as being controlled by a midwife or doctor and, because of previous trauma suffered, that influenced decisions made for this labour and birth.

*Participant 16 - If nothing else, because of my emotional state beforehand I was so overwhelmed when he was born that I was just so happy that he was finally there and that I could start the healing process of my emotions.*

Women who had experienced traumatic births previously described birthing at home, relatively unassisted, as a healing experience. These women expressed a renewed self-confidence in their own abilities, that birth occurred without interference and that they could trust their bodies to birth without intervention in the future.

*Participant 19 - It was difficult because what I was experiencing was real bonding, real attachment and how it should have been. And that was actually quite hard for me … because … I know this didn’t happen last time. And I know this is how it’s meant to be.*

The women’s retrospective attitudes toward their unplanned birth experience appeared to impact their feelings about childbirth in general. Women reflected how they previously felt about birth and how their opinions had changed, having gone through this experience.

*Participant 22 - I’d already started thinking we’re made to do this, this is what we’re supposed to do, it isn’t a medical condition.*

For women in this study the experience of unplanned out-of-hospital birth was predominantly empowering. Regardless of some negative interactions with healthcare professionals, these women developed a new perspective on birthing and their body’s capabilities, that formed or reinforced their beliefs about natural birth without medical intervention.

## Discussion

The information gathered from these women’s birth stories challenges data that suggests unplanned out-of-hospital birth predominantly occurs in multigravida women who have a precipitate birth or in women who lack antenatal care. The women interviewed discussed their knowledge, beliefs and experience of the birth process and the professional models of care associated with the hospital and unplanned out-of-hospital environment. They described not being heard, not being listened to by health professionals, and based on their previous birth experience, they communicate a lack of trust in the care provided by hospital-based care providers, specifically midwives and doctors. Women described being treated poorly and feeling unsupported. The women also discussed how birthing information they received was based on the medical professions’ assessment of risk and did not support autonomy in childbirth or respect the ability of the parent to make their own decisions.

The participants expressed a belief that in a hospital system they need to seek permission, avoid the rules of policy, or comply because policy or authorities dictated they should. They did not describe a collaborative process in decision making in either the hospital or out-of-hospital setting. This is counterintuitive to the intentions of woman-centred care. The women’s stories highlight the value of women-centred care, to better listen to what women want rather than impose clinical protocols or guidelines on them. These narratives also show that the women recognise that although the notion of women-centred care is promoted there are limitations to the ability of midwives being advocates for them.

It may be assumed that the doctor or midwife is the ‘expert’ in birth processes because they have academic and professional expertise. However, there is a growing counter narrative which purports that the authoritative knowledge held by medical professionals fails to take account or encompass birthing mothers embodied and intuitive knowledge/s [15]. This is often perceived by women during pregnancy, labour and birth as patronising, and as an imposition of an unwarranted hierarchy of power [26, 27]. The women’s narratives presented above aligns with this counter-narrative.

This research project has revealed examples of hitherto hidden or unvoiced experiences of women during unplanned out-of-hospital birth. The participants described tensions between themselves and care providers predominantly arising from what constituted and who possessed authoritative knowledge. Women described finding themselves experiencing primal, embodied and intuitive knowledge during the birth of their child which was often at odds with the messages, instructions and demands they received from doctors and midwives.

The use of the term ‘authoritative knowledge’ in this context differs from the knowledge of professionals in authority positions, such as doctors or midwives. The term instead describes how laypersons involved in a particular situation acquire their knowledge through practise or experience, convey that knowledge to others, and use it to inform decisions [28]. At times, the woman’s authoritative knowledge may differ from the dominant medical discourses held by healthcare professionals. Indeed, studies indicate that women and the medical profession often have divergent views on the nature and process of childbirth and the context in which it is viewed [29–31].

Relatedly, Ellingson & Buzzanell [32] identify the asymmetrical relationship between health professionals and patients. The healthcare professional is often positioned as the holder of knowledge, authority and power, whereas the patient can be situated as passive and ignorant. However, to prepare for childbirth many women actively acquire information they believe will equip them with agency during the birthing process. Women also describe not wanting to appear *‘naïve’* or for their ideas and opinions to be marginalised by healthcare professionals. Some women interpret the healthcare professionals’ verbal and non-verbal communication as *‘patronising’* or not being treated *‘like you’re a person; you’re just a thing’.* Finally, many women did not feel respected by the professionals or in control of the birthing process and felt that their embodied and experiential authoritative knowledge was not valued.

### Limitations

This study was conducted in Queensland, Australia and reflects the Queensland maternity system, and the cultural perceptions of women birthing in Queensland, Australia. The recruitment approach suggests that the women who participated in the study were interested in the subject. Although increasing in frequency, unplanned out-of-hospital birth occurs infrequently in Queensland, the collected narratives therefore represent a sample of 22 women and are not necessarily representative of all women.

## Conclusion

Notwithstanding the numerous challenges that influenced the decision to access maternity care when labour commenced, many of the participants interviewed communicated an overwhelming need and determination to remain autonomous during their birth experience. These women expressed an embodied confidence to birth without intervention and trusted their instincts during the birth process. One way to maintain autonomy for these women was hospital avoidance, a decision largely informed by their previous birthing experiences. The research reported here offers a new perspective on why some women experience ‘unplanned’ out of hospital births and runs counter to the dominant medicalised narrative that unplanned out-of-hospital birth are predominately triggered by multiparity and births that are precipitate in nature. It also highlights the possibility that women may increase their chances of having an unplanned out-of-hospital birth because of the actions they take in response to feeling unsupported by and pressured within the hospital and medicalised system. The findings in this study thus provide an opportunity for healthcare policymakers and professionals to reflect upon what is defined as ‘active labour’, when and how women can be admitted into hospital for childbirth, ante-natal education, the power of professionals’ behaviour and communication during birth, to listen deeply and respond to women’s child birthing preferences, and value women’s embodied authoritative knowledge.

## Data Availability

The identifiable data that support the findings of this study are not publicly available. Restrictions apply to the availability of the non-identifiable data. Contact the principle author Dr. Belinda Flanagan for further details BFlanaga@usc.edu.au

## Abbreviations

BBA: Birth Before Arrival
